# Effect of implantation site of the His bundle pacing leads on pacing parameters: a single-center experience

**DOI:** 10.1186/s12872-020-01842-1

**Published:** 2021-02-24

**Authors:** Oushan Tang, Haoliang Zhou, Caidi Yuan, Yinhong Cheng, Jin Lv

**Affiliations:** 1 Department of Cardiology, The Second Hospital Affiliated to ShaoXing University, 123 Yan‘an Road, Shaoxing, 312000 Zhejiang Province China; 2 Department of Ultrasound, The Second Hospital Affiliated to ShaoXing University, 123 Yan‘an Road, Shaoxing, 312000 Zhejiang Province China

**Keywords:** His bundle pacing, Location, Sensing

## Abstract

**Background:**

HB pacing is a promising approach to achieve physiological pacing, but its efficacy and long-term effects require further validation. In current study, we deemed to investigate the effect of the His bundle pacing (HBP) lead location on pacing parameters.

**Methods:**

2D echocardiography imaging was performed after successful implantation, according to which the patients were divided into groups A (whose His lead tips were at the atrial side) and B (whose His lead tips were at the ventricular side). The capture thresholds, sensing values, and H-V intervals between the two groups were compared.

**Results:**

Thirteen patients were in group A and 16 patients were in group B. The average capture thresholds during, 1 month, and 1 year after operation were 1.20 ± 0.34, 0.69 ± 0.29, and 0.92 ± 0.80 V/0.5 ms for group A and 1.14 ± 0.43, 0.81 ± 0.39, and 0.98 ± 0.59 V/0.5 ms for group B, respectively. The difference between the two groups was not significant. The threshold values in both groups decreased significantly in 1 month and slightly increased in 1 year. The sensing values of group A were 1.87 ± 0.82, 1.95 ± 0.76, and 1.88 ± 0.75 mV, while those of group B were 4.53 ± 1.37, 4.69 ± 1.38, and 4.59 ± 1.42 mV. The difference among the three time points was not significant. However, the sensing values in group A were consistently significantly lower than those in group B. The HV interval in group A was significantly longer than that in group B.

**Conclusions:**

The implantation site of HBP leads has a significant effect on sensing values for that His leads crossing the tricuspid annulus toward the ventricle are associated with higher sensing values, compared to a more proximal location. Meanwhile, lead location has no evident effect on capture thresholds that is improved significantly shortly after operation.

## Backgroud

Traditional right ventricular pacing is safe and easy to implant. However, it may deteriorate cardiac function and increase the incidence of atrial fibrillation after long-time application [1–3]. His bundle pacing (HBP) conduction tract is a physiologic pacing site, which truly realizes cardiac physiological synchronization. With the development of tools and improvement of the technique in recent years, the acceptance of HBP has increased. However, HBP is considered to be associated with low sensing values and uncertain long-term results, which are obstacles in its widespread use [4]. Based on clinical practice, the implantation site of the His lead may have an impact on pacing parameters, for which implanting the leads to the distal site of the His bundle can significantly increase the sensing values. To date, studies on this field are few. In the present study, 2D echocardiography imaging was used to detect the implantation site of leads, and the effect of lead location on pacing parameters was explored. Long-term safety was also evaluated based on follow-up.

## Methods

### Patient selection

We analyzed all cases of HBP in our hospital from January 2016 to June 2018. All patients had permanent atrial fibrillation with atrioventricular nodal block and clinical symptoms. The selection criteria were as follows: (1) Compliance with selective and nonselective pacing criteria for HBP in 2017 [5] and (2) standardized follow-up data after operation. The exclusion criteria were as follows: (1) Compliance with left bundle branch block or H-V conduction disturbances, (2) Absence of clear H wave potential and uncertain nonselective pacing during operation, (3) dislocation of the lead within 1 month after operation, and (4) severe multiple organ diseases. The study was approved by the hospital institutional review board, and informed consent was obtained from each patient.

### Implantation technique

His bundle leads were implanted through a method similar to that of Sharma [6]. All patients were implanted with a 4.1-Fr bipolar active fixation lead (Select Secure, model 3830, Medtronic Inc., Minneapolis, MN, USA), assisted by a C315 sheath (C304 sheath if necessary), and some cases were mapped with double leads. H wave to ventricular wave interval (HV interval) was measured using His intraluminal mapping. A Medtronic 2090 programmer was used to measure the parameters. The last unipolar parameters were recorded before the permanent pacemaker was connected. The pulse width was set to 0.5 ms, and 5.6 mV was recorded if the sensing values were over 5.6 mV. Overdrive pacing at 110 times/ min was used to rule out patients with H-V conduction disturbances.

### Echocardiography mapping

Echocardiography was done both before and within one-week after the operation in the ward using a GE log9 ultrasonic device. First, we completed the routine measurement of echocardiography parameters, such as left ventricular ID, left atrium ID, EF, tricuspid regurgitation and so on. Then we managed to find the tip of His lead during the first week echo-reexamine. We detected the fixed position of the His lead tip by combining the apex four-chamber mapping with the parasternal four-chamber mapping. Tricuspid annulus was defined as following the ESC/EACTS guidelines for the management of valvular heart disease [7]. Attachment point of tricuspid septal valve was used as the measurement mark of the tricuspid annulus. The distance from the tip of the lead to the mark was measured, and the average value of three measurements was recorded. The tip at the atrial side of the annulus was marked negative and the ventricular side positive. We utilized following methods to decrease error as much as possible: eco information was done by the same clinical specialist using the same type of device; data was read at diastolic phase; three data were collected, and the average value was applied for every patient.

### Follow-up

Patients were followed up in the clinic at 1, 3, 6, and 12 months after operation and half yearly thereafter. During the follow-up, electrocardiogram (ECG), echocardiography, brain natriuretic peptide test, and other examinations were completed as required, and New York Heart Association failure classification grade was assessed. The capture thresholds, sensing values, impedance, and pacing ratio were measured using a Medtronic 2090 programmer. The pacemaker was programmed to AAI mode to obtain the His lead parameters if possible, and the sensing value was measured using a unipolar. Far-field atrial over-sensing was excluded via different sensing sensitivities to obtain the real sensing values. If the capture threshold was below 0.5 V/0.5 ms, 0.5 mV was recorded, and sensing values above 5.6 mV was recorded as 5.6 mV.

### Statistical analysis

Statistical analysis was carried out using SPSS 20.0 software. Continuous data are presented as mean ± standard deviation, while counting data are presented as %. Significant differences between groups were detected by either t-test or chi square test. The statistical significance was defined as a *p*-value < 0.05.

## Results

### Patient characteristics

Between January 2016 and June 2018, a total of 38 cases of HBP were analyzed. Data of 29 cases, including 23 men and 6 women, aged 65–90 years, with an average age of 76 ± 7.15 years were applied for current study. The baseline characteristics of the patients are shown in Table 1. Nine cases were ruled out for the following reasons: dislocation of His leads within one week (one case), critical cerebral infarction happen in one month (one case), and for either no clear H wave potential or uncertain HBP (7 cases). Dual-chamber pacemakers were implanted for all patients. His leads were placed on the atrial pole and the right ventricular septal or left bundle branch leads to the ventricular pole as back up. The follow-up data are shown in Table 2.

**Table1:** Baseline characteristics of the all patients

Age	Sex (male)	Hypertension	Diabetes	Cerebral infarction	Valvular disease	Coronary artery disease	LVEF (%)	LA (mm)	LVIDd (mm)	NYHA
76.1 ± 7.2	23(79.3%)	24(82.8%)	5(17.2%)	6(20.7%)	3(10.3%)	7(24.1%)	66.4 ± 12.1	47.4 ± 6.3	52.8 ± 7.9	2.3 ± 0.54

LVEF, left ventricular ejection fraction; LA, left atrium; LVIDd, left ventricular end diastolic dimension; NYHA, New York Heart Association

**Table 2:** Patients’ pacing parameters

PT#	Month	AT implant				One month			HBP	One year			Lead tip location (mm)
		Threshold v/0.4 ms	Sensing mv	Impedance (Ω)	h-V (ms)	Threshold V/0.4 ms	Sensing mv	Impedance (Ω)	Type*	Threshold v/0.4 ms	Sensing mv	Impedance (Ω)	
1	42	1.2	6.8	656	46	1.20	5.6	560	2	1.50	5.6	550	6.7
2	42	1.2	1.4	670	55	0.75	1.4	567	1	0.75	1.4	546	− 1.4
3	40	1.0	2.4	715	39	0.50	2.4	675	1	0.50	1.8	656	− 1.0
4	40	1.4	1.4	743	55	0.50	1.4	463	1	0.50	1.4	440	− 3.3
5	39	1.4	2.8	887	51	0.50	2.4	782	2	0.50	2.4	796	− 3.4
6	37	1.8	1.0	559	50	0.50	1.2	456	1	0.50	1.2	440	− 4.0
7	37	1.4	3.4	682	48	1.50	3.4	590	2	2.00	3.4	576	3.0
8	36	1.2	4.5	774	35	0.75	5.6	564	2	0.75	5.6	574	6.5
9	12	1.2	2.2	715	40	1.50	2.4	978	1	3.50	1.7	1026	− 2.4
10	13	1.2	2.2	696	60	0.75	3.0	450	1	0.75	3.2	443	− 3.0
11	32	1.0	5.0	695	34	1.00	5.6	674	2	1.00	5.6	653	8.0
12	32	1.0	0.9	682	51	1.00	1.2	540	2	1.00	1.4	542	− 2.3
13	26	1.8	3.5	967	36	1.75	2.4	768	2	1.75	2.0	765	3.0
14	26	1.0	1.8	610	50	0.75	1.8	438	1	0.75	1.7	432	− 1.3
15	26	0.6	1.6	578	42	0.50	1.2	453	1	0.50	1.2	440	− 3.9
16	25	1.0	3.6	583	36	0.75	4.8	453	1	2.00	2.4	967	7.9
17	25	0.8	3.5	570	31	0.75	5.6	431	1	0.75	5.6	440	7.0
18	21	0.8	1.8	590	48	0.50	2.4	540	1	1.00	1.8	520	− 5.9
19	23	1.0	6.8	823	41	0.50	5.6	673	2	0.50	5.6	673	6.7
20	21	1.7	3.8	614	55	0.75	3.4	458	1	0.50	3.4	450	− 5.8
21	19	0.5	5.6	630	31	0.75	5.6	540	2	0.75	5.6	550	9.2
22	16	0.8	4.5	585	35	0.50	4.5	430	1	0.50	4.5	425	5.0
23	16	2.0	5.6	676	38	0.50	5.6	454	1	0.50	5.6	443	6.8
24	13	0.6	4.0	884	42	1.00	4.0	756	2	1.75	4.0	967	5.3
25	12	1.0	1.0	574	50	0.50	1.2	455	1	1.00	1.2	396	− 3.4
26	13	1.2	1.6	580	36	0.5	1.6	430	1	0.5	1.8	430	1.0
27	12	1.0	5.6	690	36	0.5	5.6	467	2	0.5	5.6	450	7.3
28	12	1.8	3.5	610	32	0.5	4.8	540	1	0.5	5.0	556	7.3
29	12	1.0	5.5	518	33	0.5	5.6	430	1	0.5	5.6	424	7.2

*Type 1, selective HBP; Type 2, non-selective HBP

### Parameters in the two groups

Among 29 patients, 13 cases (group A) had the tips of the His leads at the atrial side, the 16 cases (group B), including the three cases with crossing annular within 3 mm, had the tips of the His leads at the ventricular side. The average distance of the tips of His leads to the annulus was − 3.16 ± 1.54 mm in group A and 6.12 ± 2.21 mm in group B. The mean intraoperative capture threshold for all cases was 1.16 V that decreased significantly to 0.76 V at 1 month after the operation. The average sensing value was 3.36 mV during operation and 3.49 mV at 1 month, with no significant change. The capture threshold, sensing values and impedance of groups A and B are shown in Table 3. The capture thresholds in both groups decreased significantly at 1-month follow-up after operation (*p* = 0.002) and increased slightly at 1-year follow-up. However, the difference between the two groups at the same period was not significant. Meanwhile, the average sensing value in group A was 1.87 ± 0.82 mV and that in group B was 4.53 ± 1.37 mV during operation. The sensing value in group A was significantly lower than that in group B (*p* < 0.01); no significant changes were observed during 1-month and 1-year follow-up in the same group. As for the impedance, no significant change was found between the two groups.

**Table 3:** Comparison of the pacing parameters between groups A and B

Time schedule	Capture (V/0.5 ms)		Sensing value (mV)		Impedance	
	A (n = 13)	B (n = 16)	A (n = 13)	B (n = 16)	A (n = 13)	B (n = 16)
During operation	1.20 ± 0.34	1.14 ± 0.43	1.87 ± 0.82	4.53 ± 1.37*	626 ± 91	690 ± 123
1 month	0.69 ± 4.44**	0.81 ± 0.39*	1.95 ± 0.76	4.69 ± 1.38*	529 ± 163	528 ± 117
1 year	0.92 ± 0.80	0.98 ± 0.59	1.88 ± 0.75	4.59 ± 1.42*	522 ± 181	574 ± 178

*p < 0.05 versus group A

**p < 0.05 1 month versus during operation

### Correlation between sensing value and location sites

**Fig. 1:**
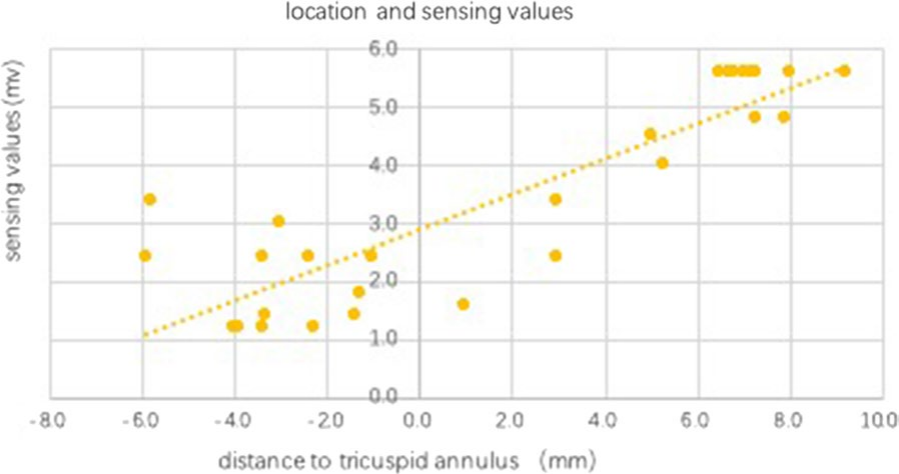
Lead location and sensing value. The sensing value and the distance to the annulus for each patient were recorded and described using a scatter plot

The lead location and sensing value were further described using a scatter plot (Fig. 1), showing that the majority of the sensing values in group A were below 3.0 mV. No special relationship was detected between the sensing value and the distance to the annulus, and the distribution of scatter points was observed. In group B, the farther the distance to the annulus, the higher the sensing value, and the sensing values and the distance of lead tips to the annulus had a linear correlation. R wave amplitudes in a large proportion of cases (9/16) exceeded the program-controlled upper limitation of 5.6 mV.

### Correlation between H-V interval and location distance

**Fig. 2:**
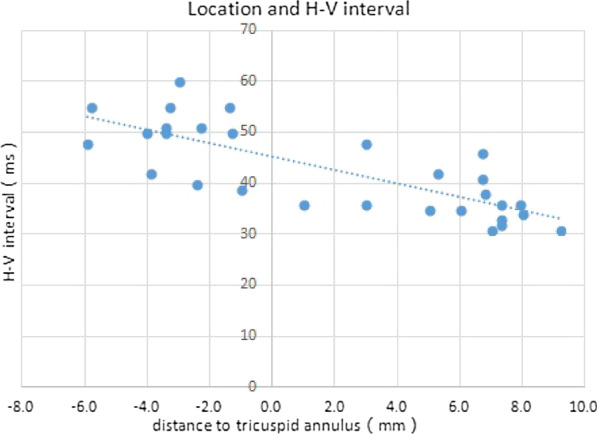
Lead location and H-V interval. Negative meant leads located at atrium side. H-V intervals scatted around 50 ms. Positive meant leads located at ventricular side, HV intervals had a linear distribution

H-V interval was measured during operation for all patients. 28 patients had HV interval between 30 to 55 ms with one except 60 ms. Overdriving pacing at 110 times/min was done to rule out HV conduction disturbances. When we compared the average H-V interval between group A and B, we found Intraoperative HV interval in group A was significantly longer than that in group B (*p* < 0.01) ( 49.69 ms vs 36.56 ms). If three cases of proximal annulus were excluded in group B, the remaining 13 cases (Distal group) had the lead tips positioned at the ventricular side of the annulus by more than 5 mm. Then the HV interval was shortened to an average of 35.77 ms, which indicates that the more distal the His lead located, the shorter the HV interval. Refer to the scatter plot (Fig. 2), a correlation between the lead location distance and H-V interval could be observed.

### Parameters between selective and non-selective cases

At 1-month follow-up after operation, the proportion of sHBP in group A was 84.6% (11/13), which was significantly higher than that in group B 43.8% (7/16) (*p* < 0.01), as shown in Table 4. Among all 29 cases, there were altogether 18 sHBP and 11 nsHBP cases. R wave in the nsHBP was 4.27 ± 1.78 mv which was significantly bigger than that of the SHBP 3.16 ± 1.74 mv. Capture threshold in the nsHBP was 0.95 ± 0.47 v/0.5 ms which was similar to that of the SHBP 0.78 ± 0.35v/0.5 ms. However, when we set working potential at over 2.5 mv/0.5 ms, there was no selective pacing case.

**Table 4:** HV interval and S-HBP ratio

Time schedule	HV interval (ms)			S-HBP ratio	
	A (n = 13)	B (n = 16)	D (n = 13)	A (n = 13)	B (n = 16)
During operation	49.69 ± 6.22	36.56 ± 4.44*	35.77 ± 4.41	–	–
1 month	–	–		11 (84.6%) **	7 (43.8%)

*p < 0.01 versus group A

**p < 0.01 1-month versus during operation

### Case analysis of the lead location image and ECG

**Fig. 3:**
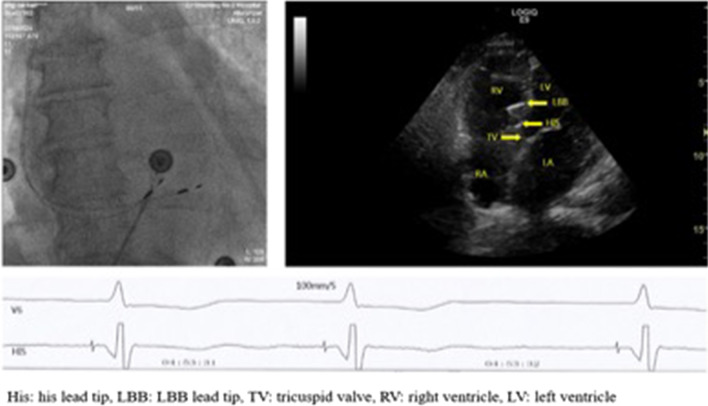
DSA image, 2D-Echocardiography mapping and electrophysiological intracavity map for patient case 29

**Fig. 4:**
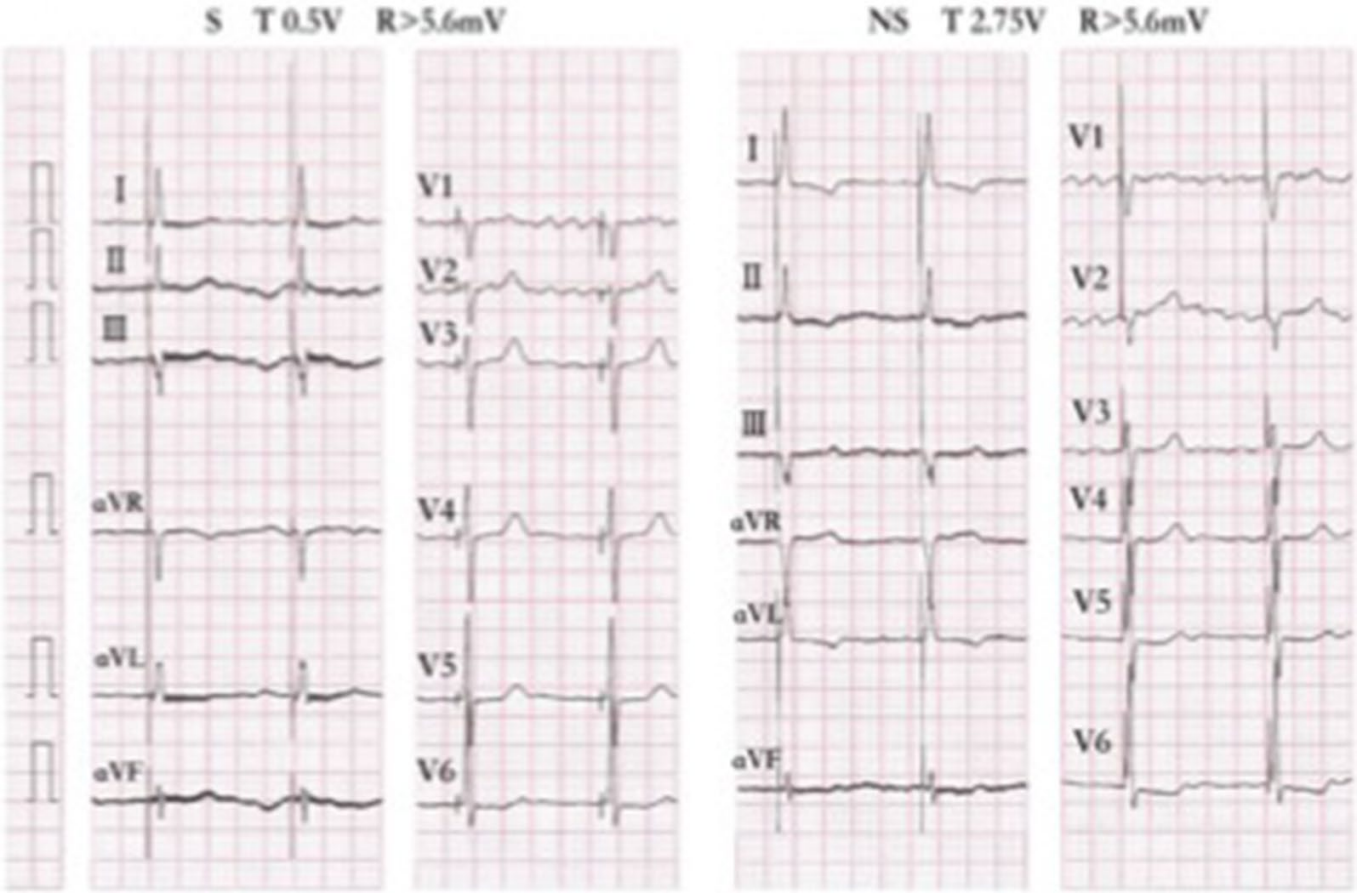
Selective and non-selective pacing for patient case 29

Case 29 is an 85-year-old woman, with significant bradycardia and heart failure. She was implanted with His bundle and left bundle branch leads. HV interval was 33 mV. The intra-operative capture threshold of the His lead was 1.0 V, and the sensing value was 5.5 mV. The selective capture threshold was 0.5 V, and the nonselective one was 2.75 V at 1 month. During follow-up, output voltage was programmed to 2.5 V, and the sensing value was over 5.6 mV. 2D echocardiography mapping showed that the His lead tip was located at the ventricular side, 7.2 mm away from the attachment point of tricuspid septal valve. (Figs. 3 and 4).

### Problems in programming

Very low sensing value were observed in group A during early follow-ups. When we set the sensing sensitivity to 0.18 mV, we obtained a sensing value of 0.35–0.5 mV in 4 cases (4/13). If the sensing sensitivity decreased to 0.5–0.7 mV, we obtained a better sensing value of 1.2–2.8 mV in the same patients. It indicated His leads could sense atrial potential in advance under high sensitivity. After the sensing sensitivity was decreased, far-field sensing of the atrium was avoided. No similar phenomenon was found in group B. Furthermore, the sensing values of the patients whose lead tips exceeded the annular by more than 5 mm were significantly improved, and those of 9 patients (9/16) were over 5.6 mV.

### Findings during follow-up

Twenty-nine patients were followed up for 12–42 months, with an average of 25 months. Clinic symptoms were improved for all patients and no serious tricuspid regurgitation was recorded. Twenty-six patients (89.66%) had improved or stabilized lead parameters during follow-up, of which 23 patients (79.31%) had capture threshold less than or equal to 1.0 mV at 1 year. During the follow-up 1 year after the operation, the capture threshold of three cases (9, 16, and 24) increased gradually to over 1.0 V, whereas the sensing value decreased. We found by reviewing the intraoperative lead X-ray positioning and echocardiography images that the leads were obliquely fixed in cases 16 and 24. However, we did not observe this phenomenon for case 9.

## Discussion

His bundle connects the atrioventricular node with the left and right bundle branches. It originates from the atrioventricular node and passes through the membranous interventricular septum. The average length of the bundle is 20 mm. Any part of the bundle can be located for pacing. However, reports on the location of HBP leads and the effect of the location site on pacing parameters are few. Correa de Sa et al. [8] reported a case of an 81-year-old woman who had been implanted with HBP leads 2 years before death. Anatomical observations revealed that the tip of the lead was located at the atrial side of the tricuspid annulus. Vijayaraman et al. [9] assessed a 42-year-old man with His lead via 2D echocardiography and CT imaging. The tip of the His lead was implanted at the atrial side of the tricuspid annulus. Those authors suggested that atrial side HBP can reduce the impact on the tricuspid valve, which is considered an advantage of HBP. Alexander et al. [10] studied five canine hearts in vitro to determine whether the location of the lead affects the pacing parameters. They analyzed the relationship between lead location sites and pacing parameters and ECG morphology at four different sites of the His bundle from the proximal to the distal locations. The results showed that the difference between the proximal (area 2) and distal (area 4) locations on capture threshold was not significant, but the proportion of selective pacing in the former was higher than that in the latter. Of note, none of the above-mentioned studies described influence of the location of the His lead on sensing values. The finding of our current work, that is, that the implantation site of HBP leads has a significant effect on the sensing values may contribute to limited literature available on the effects of HBP implantation site.

In the present study, we selected carefully 29 cases according to 2017 standard definitions of HBP [5]. The location sites of His leads were determined by 2D echocardiography imaging. According to the relationship between the tips of the His leads and the tricuspid annulus, the patients were divided into groups A (whose tips were on the atrial side) and B (whose tips were on the ventricular side). The difference in capture threshold between the two groups at the same time was not significant, and the proportion of selective pacing was higher in group A than in group B, which is consistent with the aforementioned canine study in vitro. Meanwhile, the sensing values were significantly higher in group B than those in group A. With the leads crossing to the ventricular side, especially in patients with crossing annulus over 5 mm, the sensing values were over 4 mV, suggesting that distal HBP can solve the problem of low sensing values. The compared HV interval between the two groups suggests that the HV interval was shortened after the leads crossed the annulus. The average HV interval in group B was 36 ms, which was significantly shorter than the 49 ms interval in group A. In the more distal group (D), the HV interval was further shortened to 35 ms, suggesting that the conduction of the His bundle from the proximal to the distal location was approximately 15 ms.

In practice, it is technically difficult to determine whether the His lead has crossed the annulus during surgery by using DSA images. Our experience from current study suggested that it can be helpful to determine whether distal HBP is achieved by measuring sensing values and HV interval during surgery. We suggest the use of sensing values over 4.0 mV combined with HV interval shorter than 40 ms as a reference index for distal HBP.

The capture threshold of HBP pacing is considered higher than that of conventional ventricular pacing. Vijayaraman et al. [4] reported 75 cases with an average implantation capture threshold of 1.35 ± 0.9 V/0.5 ms. In current study, the average intra-operative capture threshold was 1.16 ± 0.38 V /0.5 ms, which is slightly higher than the usual ventricular threshold. Short-term follow-up results showed that the capture threshold improved significantly, and the average capture threshold decreased to 0.76 ± 0.35 V/0.5 ms at 1 month. During the follow-up period, approximately 90% of the cases remained stable, and approximately 80% of them had a chronic threshold of less than 1.0 V. Combined with case 29 analysis, the capture threshold of HBP may be similar to that of the ventricular pacing but with prolonged waiting time. Since the increasement of the His lead capture threshold is closely related to intra-operative lead fixation, we suggest referring to LAO 40°–45° fluoroscopy image to ensure that the His lead is vertically fixed.

We also summarized the following practice notes from our experience: the pacemaker should be programmed to AAI mode if possible to measure His lead parameters and to avoid potential interference. When the lead tip is on the atrial side, theoretically the lead can sense the potential of the atrium and the ventricular, and the atrial potential is always before that of the ventricular. When the pacemaker senses the atrial one, it would no longer sense the ventricular one, which may lead to a false low sensing value. When sensing is measured, the sense sensitivity should be adjusted to avoid excessive sensitivity setting, leading to far-field atrial sensing, which is always mistaken for a low His sensing value. We noticed this phenomenon happened in four cases in group A, which was related to the position of the His lead and potential size of atrium. It is believed that atrial cross sensing might lead to cardiac arrest if the patient is dependent on pacing. Although up to now no systemic study has been reported on this topic, we suggest plant the lead to a distal site to avoid this problem.

An important concern on HBP is the long-term safety especially for sHBP in the atrium. We ruled out patients with LBBB or wide QRS morphology and used overdriving pacing during operation to reduce this problem. We also planted a second ventricular lead for safety. Non-selective pacing which means capture both His-purkinje system and surrounding RV myocardium might be safer. In our study, the sensing value was better in non-selective group; the capture threshold was similar between the two groups. Actually, for all the cases worked under non-selective pacing mode at working output potential over 2.5 v/0.5 ms, no case was recorded with new conduction disturbances during the follow-up period.

In summary, we have two major findings in this study. First, the sensing values of His lead-crossed tricuspid annulus were improved significantly, and the farther distal the lead is, the better the sensing value. Second, the correlation between the location of the leads and capture thresholds was not significant. The capture thresholds decreased significantly at 1-month follow-up after operation and are kept steady in the late period. Nevertheless, this work has several limitations. This is a small-scale single-center observational study with limited follow-up time. Distal HBP requires more surgical techniques than the proximal one, which restricts its widespread application. Moreover, it requires more implantation tools to simplify its operation. The long-term safety and efficacy of distal HBP still require a large sample of multicenter prospective clinical trials.

In conclusion, HBP is a promising mode of physiological pacing for future applications in patients. Further studies on the effects of implantation location on HBP leads and improvement of this technique through further clinical trials may lead to its more widespread adaptation.

## Data Availability

The datasets used and/or analyzed during the current study available from the corresponding author on reasonable request.
